# Associations between oxygen saturation Index and oxygenation index in neonates with congenital diaphragmatic hernia

**DOI:** 10.3389/fped.2024.1389062

**Published:** 2024-07-17

**Authors:** Kamal Ali, Saleh S. Algarni, Abdullah M. Alotaibi, Nemer Aljuaid, Abadi Ghazwani, Saad Alshreedah, Naif Alotaibi, Ibrahim Alanazi, Mashael Almutairi, Manal Althubaiti, Faisal Alsehli, Ahmed Alwatban, Saif Alsaif

**Affiliations:** ^1^ Neonatal Intensive Care Department, King Abdulaziz Medical City-Riyadh, Ministry of National Guard Health Affairs, Riyadh, Saudi Arabia; ^2^ King Saud bin Abdulaziz University for Health Sciences, Riyadh, Saudi Arabia; ^3^ King Abdullah International Medical Research Center, Riyadh, Saudi Arabia

**Keywords:** congenital, diaphragmatic, hernia, oxygen index, oxygen saturation index, correlations

## Abstract

**Objective:**

To explore the relationship between Oxygenation Index (OI) and Oxygen Saturation Index (OSI) among infants with Congenital Diaphragmatic Hernia (CDH), both within the first 24 h after birth and in extended observations in those who survived until their surgical intervention.

**Methods:**

Seven- years retrospective review of CDH cases at a single Level III neonatal intensive care unit. The correlations of various combinations of OI-OSI pairs were assessed using the Spearman's rho Correlation Coefficient. Additionally, during the initial 24 h, the correlations between admission (first), best (lowest), highest, and mean OI and OSI values were determined. The predictive ability of the first 24 h oxygen and oxygen saturation indices for mortality and other adverse outcomes were assessed using the Area Under the Curve (AUC) analysis.

**Results:**

Thirty-seven infants with CDH were included in the analysis. A strong correlation was observed between all pairs of OI/OSI (2,289) (Spearman's rho = 0.843), matched pairs of Postductal OI/OSI (1,232 pairs) (Spearman's rho = 0.835) and the unmatched pairs of Postductal OI and Preductal OSI (1,057 pairs) (Spearman's rho = 0.852). Using the regression equations for all pairs, matched and unmatched OI/OSI pairs, we deduced that for clinically pertinent OI thresholds of 10, 15, 20 and 40, the corresponding OSI values were 5, 8, 11, and 23, respectively. Furthermore, in the first 24 h, strong correlations were evident between OI/OSI: at admission (Spearman's rho = 0.783), best OI/OSI (Spearman's rho = 0.848), and highest OI/OSI (Spearman's rho = 0.921). The most robust correlation was observed between the mean OI/OSI with a Spearman's rho of 0.928. First (AUC = 0.849), best (AUC = 0.927), highest (AUC = 0.942) and mean day 1 OI (AUC = 0.946) were all predictive of mortality. Similarly, first (AUC = 1.00), best (AUC = 0.989), highest (AUC = 1.00) and the mean OSI in day 1 (AUC = 0.978) were all predictive of mortality. All of the OIs and OSIs in day 1 except for the admission OSI (AUC = 0.683) were predictive of pulmonary hypertension. Additionally, all of OI and OSI indices in the first 24-hour except for the best day 1 OI (AUC = 0.674) were predictive of the need for rescue HFOV.

**Conclusion:**

There were a strong correlation between the OI and OSI in infants with CDH. Oxygenation indices and OSI in the first 24 h were predictive of mortality and other adverse outcomes in infants with CDH.

## Introduction

Congenital Diaphragmatic Hernia (CDH) is a birth defect characterized by the incomplete closure of the diaphragm, leading to the displacement of abdominal contents into the thoracic cavity (1). This condition can cause severe complications, including respiratory insufficiency and persistent pulmonary hypertension (PPHN) (1).

Historically, to assess respiratory status in infants with CDH, parameters like the Oxygenation Index (OI) and more recently the Oxygen Saturation Index (OSI) have been utilized (2, 3). The OI, in particular, serves as an invaluable metric for initiating inhaled nitric oxide (iNO) therapy and for considering interventions such as extracorporeal membrane oxygenation (ECMO) (4–6). Further emphasizing its significance, the OI values within the first 24 h post-birth have been identified as predictors of mortality and other adverse outcomes in infants with CDH (6–9). The formula to derive OI involves using the mean airway pressure (MAP), the fraction of inspired oxygen (FiO_2_), and the arterial partial pressure of oxygen (PaO_2_) (9). Nevertheless, the intermittent nature of OI measurements, confined to times of arterial blood sampling, restricts its efficacy for continuous bedside monitoring in infants with hypoxemic respiratory failure and CDH (4, 5, 10).

More recently, the OSI emerged as a promising non-invasive alternative. Derived from pulse oximetry readings instead of arterial blood gases, OSI provides an avenue for continuous monitoring in ventilated CDH infants (11). Its calculation relies on MAP, FiO_2_, and peripheral capillary oxygen saturation (SpO_2_) (4, 12). A retrospective analysis in newborns with hypoxemic respiratory failure identified a significant correlation between OSI and OI values (4). Additionally, the correlation between OI and OSI has recently be demonstrated in infants with CDH, suggesting its potential to substitute OI in gauging the respiratory status of CDH infants (11). Nevertheless, the OSI study in CDH infants predominantly focused on OSI values noted at admission and the highest readings within the first 24 h, leading to residual uncertainties about its full potential (11).

This study aimed to explore the relationship between OI and OSI in CDH infants, both within the critical first 24 h and in extended observations of those who survived until their surgical intervention. Furthermore, we wished to validate whether OSI, akin to OI, can be a reliable predictor of survival and potential complications, including echocardiographic evidence of pulmonary hypertension, the need for rescue high-frequency oscillatory ventilation (HFOV) and patch repair of the diaphragmatic defect at surgery. An additional objective of the study is to trace the trajectories of OI and OSI from birth until the surgical repair of the diaphragmatic defect in infants who survived and reached this critical milestone.

## Methods

We conducted a retrospective cohort study over seven years (March 2016 to March 2023) at a single Level III neonatal intensive care unit. The study comprised neonates diagnosed with CDH, requiring invasive mechanical ventilation. Participants had continuous pulse oximeter monitoring and indwelling arterial catheters from birth until either surgical intervention or death. Exclusion criteria encompassed CDH infants with associated complex congenital heart disease or major genetic abnormalities. The King Abdullah International Medical Research Centre (KAIMRC) institutional review board approved this study, and due to its retrospective nature, waived the need for informed consent.

Neonates with CDH were identified from our institution's neonatal and fetomaternal databases. Extracted data included demographic details, birth metrics, mode of delivery, CDH side, and antenatal ultrasound findings. All fetuses diagnosed antenatally underwent a thorough fetal echocardiogram at 28 weeks' gestation to check for associated congenital heart disease (CHD).

Postnatal management adhered to the department's protocol, with initial ventilation being conventional ventilation and High Frequency Oscillatory Ventilation (HFOV) serving as a rescue ventilation mode. The targeted preductal oxygen saturation and PIP were set according to the 2015 updated CDH EURO Consortium guidelines (13). Inhaled nitric oxide (iNO) was administered if echocardiographic signs indicated pulmonary hypertension, which was assessed within the first 24–48 h. The echocardiographic features of pulmonary hypertension were a right ventricular systolic pressure ≥2/3 systemic systolic pressure and right ventricle (RV) dilatation/septal displacement or RV dysfunction ± left ventricle dysfunction (14). If pulmonary hypertension was confirmed, inotropic support was initiated. During the study period, inotropic support for infants with CDH primarily consisted of dopamine, epinephrine, and norepinephrine, which were utilized as the first-line treatments for cardiac and vasopressor support. In cases of catecholamine-resistant shock, vasopressin was used. For managing hypotension that did not respond sufficiently to intravenous volume expansion and vasopressor therapy, hydrocortisone was administered. Additionally, milrinone, a pulmonary vasodilator, was used in only two infants during the study period. Surgical repair of the diaphragmatic defect took place once infants reached cardiopulmonary stability, as defined by specific clinical and mechanical ventilation criteria. In our centre surgical repair of the defect was performed when infants' clinical condition stabilized according to the following criteria: (1) blood pressure maintained at 40–50 mmhg with no more than one inotropic agent and (2) preductal oxygen saturation maintained between 85%–95% on conventional mechanical ventilation with peak inspiratory pressure at 18–20 cm H_2_O and FiO_2_ less than or equal to 50%. Our treatment protocol didn't employ extracorporeal membrane oxygenation (ECMO) as ECMO isn't available for neonatal respiratory failure at our institution.

Standard practice in our department dictated documentation of arterial blood gases, blood sampling source, concurrent oxygen saturation, and pulse oximeter location in the electronic medical records. The OI and OSI were calculated using defined formulae. All blood gases were sourced from umbilical arterial lines (Postductal), and various combinations of OI-OSI pairs were assessed. Additionally, during the initial 24 h for each neonate, metrics including admission (first), best (lowest), highest, and mean OI and OSI values were determined. Typically, infants had 4–8 arterial blood gas measurements per day, varying based on clinical stability and ventilation adjustments.

Primary outcomes included echocardiographic evidence of pulmonary hypertension, the need for rescue HFOV, need for patch repair of the diaphragmatic defect at surgery and survival to hospital discharge.

### Statistical analysis

We assessed data normality using the Shapiro-Wilk test and determined that the distribution was non-normal. Consequently, data were expressed as median (IQR) or as counts and percentages where appropriate. The relationship between OI and OSI was examined using Spearman's rho correlation coefficient. This correlation analysis encompassed both matched and unmatched pairs for the entire cohort and was further stratified based on the source of arterial blood sampling and the location of the pulse oximeter (either Preductal or Postductal). We derived OSI values using the regression equation for notable OI cut-offs of 10, 15, 20, and 40.

We evaluated the predictive abilities of the admission, mean, best, and highest OI and OSI values within the first 24 h concerning mortality, echocardiographic evidence of pulmonary hypertension, need for rescue HFOV and the need for patch repair of the diaphragmatic defect by utilizing the Area Under the Receiver Operator Characteristics (AUROC). We considered a correlation to be statistically significant if the *P*-value was less than 0.01, using a two-tailed probability test. All analyses were done using SPSS Statistics, version 26.0, by IBM Corp.

## Results

**Figure 1:**
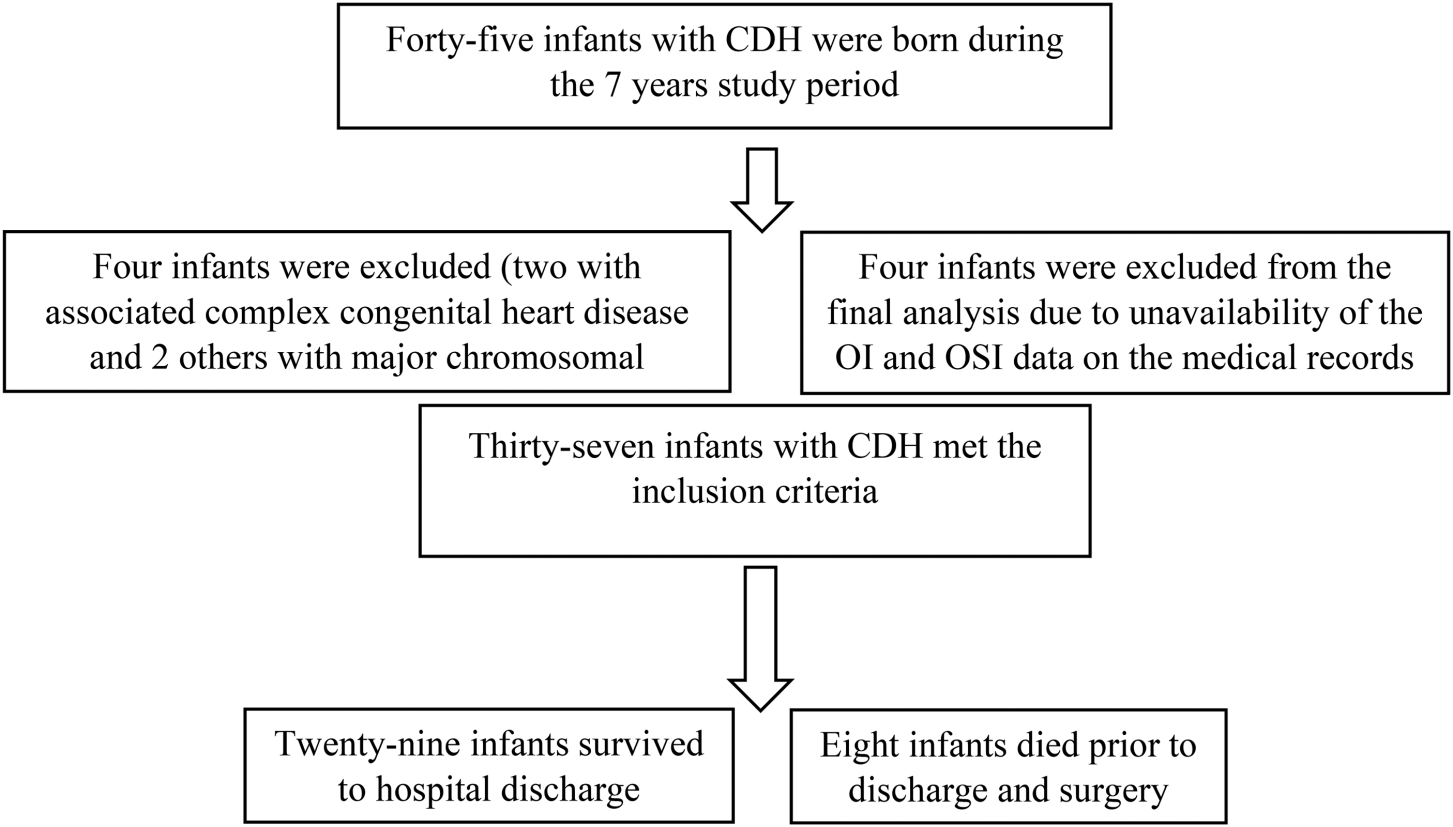
Study population.

During the study period, 45 infants with CDH were born. Of these, 2 were excluded because of associated complex congenital heart diseases, specifically one with transposition of the great arteries and another with hypoplastic left heart. Another 2 were excluded due to major chromosomal abnormalities: one had Trisomy 18 and the other had Cri-du-chat syndrome. Additionally, 4 infants were excluded due to the absence of the OI and OSI data in their electronic medical records. Ultimately, 37 infants were included in the final analysis, as depicted in Figure 1.

Table 1 above illustrates the Infants' characteristics. Of the 37 neonates studied, 54% (*n* = 20) were male and 46% (*n* = 17) female. The median birth weight was 2,600 (IQR 2,200–3,030) grams. The median gestational age of 38 (IQR: 37–39) weeks. Twenty-eight (76%) were delivered by spontaneous vaginal deliveries and 24% (*n* = 9) via cesarean section. The median Apgar scores were 5 (IQR: 4–7) at 1 min and 7 (IQR: 7–9) at 5 min. CDH was antenatally diagnosed in 84% (*n* = 31) of cases. The liver-up position was observed in 46% (*n* = 17). The CDH location was left-sided in 81% (*n* = 30) and right-sided in 19% (*n* = 7). Echocardiographic evidence of pulmonary hypertension was present in 62% (*n* = 23). Rescue HFOV was used in 73% (*n* = 27) and nitric oxide therapy in 65% (*n* = 24). Seventeen (59%) infants underwent primary repair of the diaphragmatic defect and 41% (*n* = 12) required patch repairs.

**Table 1:** Infants’ characteristics diagnosed with congenital diaphragmatic hernia.

Characteristic	(*n* = 37)
Gender	
Male	20 (54%)
Female	17 (46%)
Birth weight in gram, median [IQR]	2,600 [2,200–3,030]
Gestational age in weeks, median [IQR]	38 [37–39]
Mode of delivery	
Spontaneous vaginal delivery	28 (76%)
Cesarean - section	9 (24%)
Apgar score at 1 min, median [IQR]	5 [4–7]
Apgar score at 5 min, median [IQR]	7 [7–9]
Antenatally diagnosed CDH	31 (84%)
Liver-up position	17 (46%)
Site of CDH	
Left	30 (81%)
Right	7 (19%)
Echocardiographic evidence of pulmonary hypertension	23 (62%)
Need for rescue HFOV	27 (73%)
Nitric oxide therapy	24 (65%)
Type of surgery repair	
Primary	17 (59%)
Patch	12 (41%)
Survival to discharge	29/37 (78%)

### Correlation of all Oi/OSI (matched and unmatched pairs) from birth to surgery/mortality

**Figure 2:**
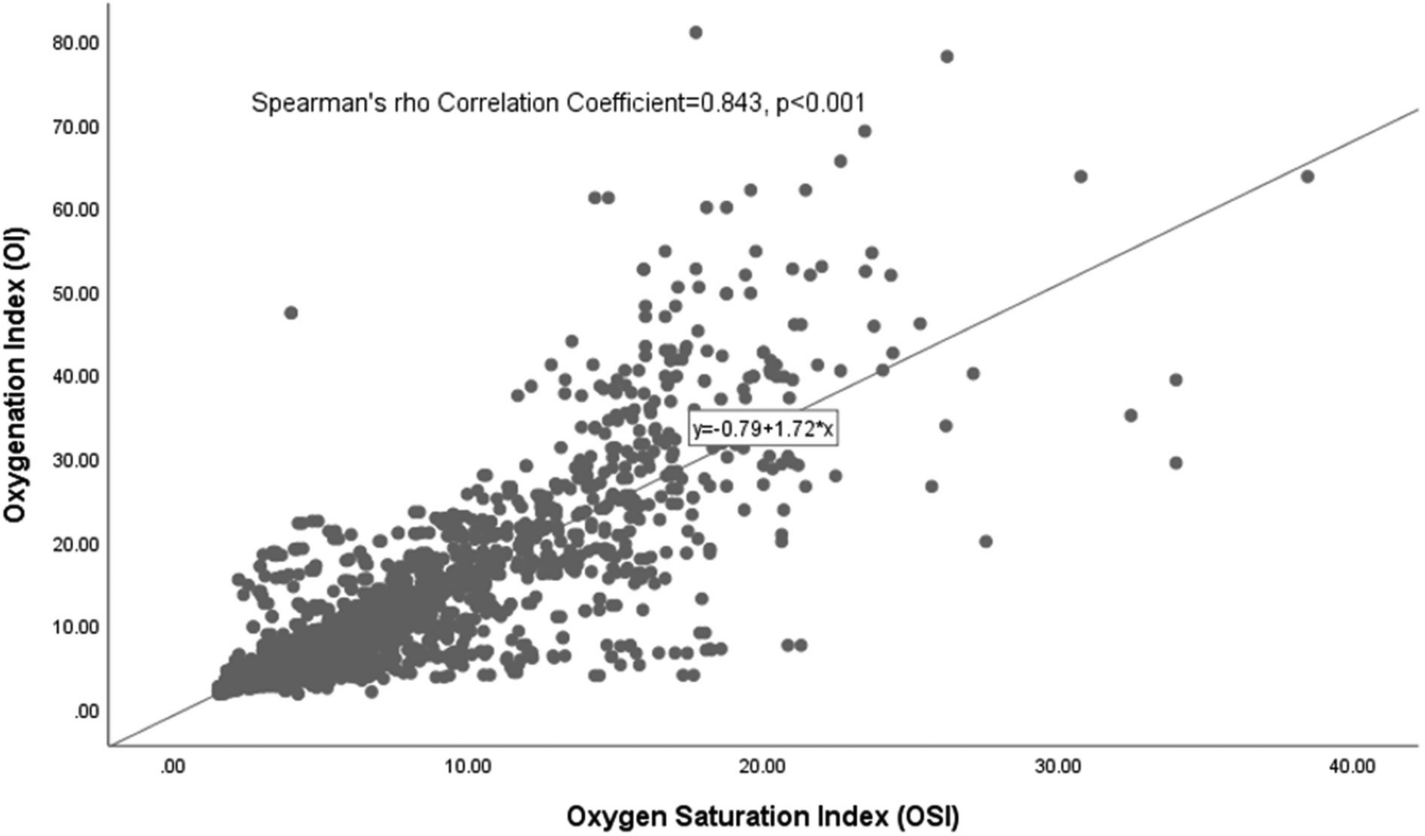
Scatter plot of the all OI/OSI pairs from birth to surgery/mortality.

For the 37 infants studied, a total of 2,289 matched and unmatched OI/OSI pairs were collected from birth until the time of either surgery or death. There was a significant correlation between OI and OSI values, with a Spearman's rho Correlation Coefficient of 0.843 (*p* < 0.001), illustrated in Figure 2. By applying the regression equation (OI = 0.79 + 1.72 * OSI), it was determined that clinically relevant OI values of 10, 15, 20, and 40 correspond to OSI values of 5.4, 8.4, 11.3, and 22.9, respectively.

### Correlation of postductal OI/OSI (matched pairs) from birth to time of surgery or death

**Figure 3:**
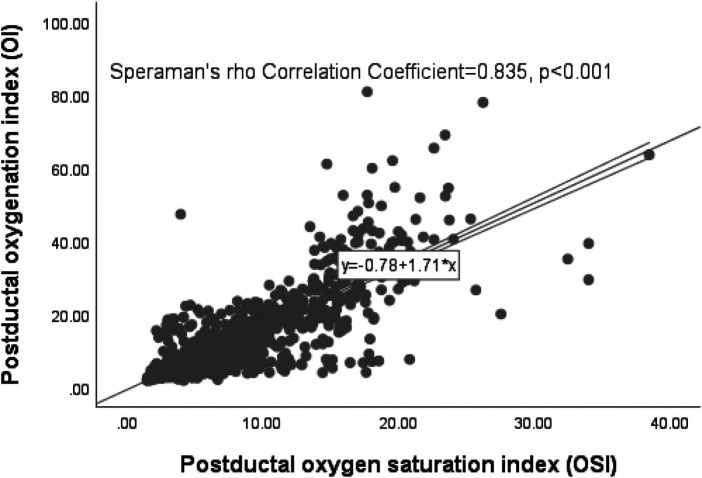
Scatter plot of the postductal OI/OSI matched pairs.

From 37 infants, 1,232 matched pairs of Postductal OI/OSI were collected from birth up to either the surgery or death. A significant correlation was found between Postductal OI and OSI, evidenced by a Spearman's rho Correlation Coefficient of 0.835 (*p* < 0.001). This relationship can be seen in Figure 3. Using the regression formula (OI = 0.78 + 1.71 * OSI), specific OSI values were calculated corresponding to clinical OI values of 10, 15, 20, and 40, resulting in OSI values of 5.4, 8.3, 11.2, and 22.8, respectively.

### Correlation of postductal OI and preductal OSI (unmatched pairs) from birth to time of surgery or death

**Figure 4:**
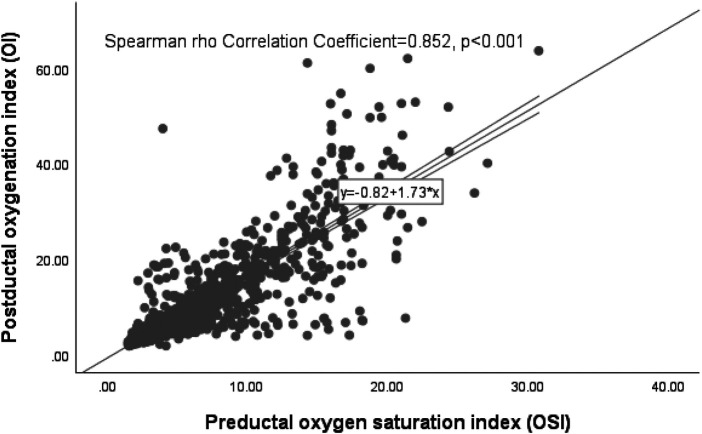
Scatter plot of the postductal OI and preductal OSI (unmatched pairs).

From the 37 infants studied, a total of 1,057 pairs of Postductal OI and Preductal OSI were collected from their birth until surgery or, in the case of non-survivors, until their mortality. A significant correlation was found between Postductal OI and Preductal OSI, with a Spearman's rho Correlation Coefficient of 0.852 (*p* < 0.001). This correlation is graphically represented in the scatter plot seen in Figure 4. Employing the regression equation from Figure 4, (OI = 0.82 + 1.73 * OSI), it was possible to determine OSI values for clinically significant OI levels. Specifically, for OI benchmarks of 10, 15, 20, and 40, the corresponding OSI readings were 5.3, 8.2, 11.1, and 22.6 respectively.

### Summary of the derived OSI values

In summary, the regression formulae presented in Figures 2–4 consistently yielded analogous derived OSI values corresponding to the clinically significant OI benchmarks of 10, 15, 20, and 40 across different OI/OSI pair categories. Specifically, for a clinically relevant OI value of 10, the corresponding OSI was 5; for the OI of 15, the corresponding OSI was 8; for the OI of 20, the corresponding OSI was 11; and for an OI of 40, the corresponding OSI was 23. These derived OSI values were rounded to the nearest whole number for the ease of interpretation. This consistency was observed irrespective of the pair type, whether considering all pairs (totaling 2,289), matched pairs (1,232 in total), or unmatched pairs (1,057 in total).

### Correlations of admission, best, highest and mean OI/OSI in the first 24 h

Tables 2, 3 present the correlations between the admission, best, mean, and highest OI and OSI values during the first 24 h post-birth. Strong correlations were evident: at admission (Spearman's rho = 0.783, *p* < 0.001), best OI/OSI values (Spearman's rho = 0.848, *p* < 0.001), and highest OI/OSI values (Spearman's rho = 0.921, *p* < 0.001). The most robust correlation was observed for the mean OI/OSI values, with a Spearman's rho of 0.928 (*p* < 0.001).

**Table 2:** Correlations of the admission and best OI/OSI in the first 24 h.

			First day 1 OI	OSI at admission	Best day 1 OI	Best day 1 OSI
Spearman's rho	OI at admission	Spearman's rho correlation coefficient	1.000	0.783^a^	0.685^a^	0.775^a^
		sig. (2-tailed)	.	<0.001	. <0.001	<0.001
		*N*	37	37	37	37
	OSI at admission	Spearman's rho correlation coefficient	0.783^a^	1.000	0.719^a^	0.868^a^
		sig. (2-tailed)	<0.001	.	<0.001	<0.001
		*N*	37	37	37	37
	Best day 1 OI	Spearman's rho correlation coefficient	0.685^a^	0.719^a^	1.000	0.848^a^
		sig. (2-tailed)	<0.001	<0.001	.	<0.001
		*N*	37	37	37	37
	Best day 1 OSI	Spearman's rho correlation coefficient	0.775^a^	0.868^a^	0.848^a^	1.000
		sig. (2-tailed)	<0.001	<0.001	<0.001	.
		*N*	37	37	37	37

OI, oxygenation index; OSI, oxygen saturation index.

^a^ Correlation is significant at the 0.01 level (2-tailed).

**Table 3:** Correlations of the highest and mean OI and OSI in the first 24 h.

			Highest day 1 OI	Highest day 1 OSI	Mean day 1 OI	Mean day 1 OSI
Spearman's rho	Highest day 1 OI	Spearman's rho correlation coefficient	1.000	0.921^a^	0.968^a^	0.933^a^
		sig. (2-tailed)	.	<0.001	<0.001	<0.001
		*N*	37	37	37	37
	Highest day 1 OSI	Spearman's rho correlation coefficient	0.921^a^	1.000	0.902^a^	0.969^a^
		sig. (2-tailed)	<0.001	.	<0.001	<0.001
		*N*	37	37	37	37
	Mean day 1 OI	Spearman's rho correlation coefficient	0.968^a^	0.902^a^	1.000	0.928^a^
		sig. (2-tailed)	<0.001	<0.001	.	<0.001
		*N*	37	37	37	37
	Mean day 1 OSI	Spearman's rho correlation coefficient	0.933^a^	0.969^a^	0.928^a^	1.000
		sig. (2-tailed)	<0.001	<0.001	<0.001	.
		*N*	37	37	37	37

OI, oxygenation index; OSI, oxygen saturation index.

^a^ Correlation is significant at the 0.01 level (2-tailed).

### Adverse neonatal outcomes

Of the 37 infants included in the study 22% did not survive, 62% had echocardiographic evidence of pulmonary hypertension, 73% required rescue HFOV and 41% of those who underwent surgery required a patch repair of the diaphragmatic defect (Table 4). The admission OI (AUC = 0.849, *p* = 0.003), best day 1 OI (AUC = 0.927, *p* < 0.001), highest day 1 OI (AUC = 0.942, *p* < 0.001) and mean day 1 OI (AUC = 0.946, *p* < 0.001) are all predictive of mortality. Similarly, the admission OSI (AUC = 1.00, *p* < 0.001), best OSI in day1 (AUC = 0.989, *p* < 0.001), highest OSI in day 1 (AUC = 1.00, *p* < 0.001) and the mean OSI in day 1 (AUC = 0.978, *p* < 0.001) were all predictive of mortality.

**Table 4:** Area under the curve using first, best, highest and mean OI/OSI in day 1 for prediction of mortality, PHT, need for rescue HFOV and patch repair at surgery.

Variable	AUROC (95%CI), Mortality (8/37 = 22%)	AUROC (95% CI) PHT (23/37 = 62%)	AUROC (95% CI) rescue HFOV (27/37 = 73%)	AUROC (95% CI) patch repair 12/29 = 41%)
Admission (first) day 1 OI	0.849 (0.724–0.974), *p* = 0.003	0.736 (0.570–0.902), *p* = 0.017	0.830 (0.679–0.980), *p* = 0.002	0.642 (0.431–0.853), *p* = 0.199
Best day 1 OI	0.927 (0.831–1.0), *p* < 0.001	0.786 (0.639–0.932), *p* = 0.004	0.674 (0.499–0.849), *p* = 0.108	0.647 (0.442–0.852), *p* = 0.184
Highest day 1 OI	0.942 (0.859–1.0), *p* < 0.001	0.761 (0.607–0.915), *p* = 0.009	0.880 (0.765–0.994), *p* < 0.001	0.652 (0.441–0.863), *p* = 0.170
Mean day 1 OI	0.946 (0.868–1.0), *p* < 0.001	0.773 (0.624–0.923), *p* = 0.006	0.815 (0.677–0.952), *p* = 0.004	0.623 (0.404–0.841), *p* = 0.268
Admission (first) day 1 OSI	1.000 (1.000–1.00), *p* < 0.001	0.683 (0.513–0.854), *p* = 0.065	0.924 (0.839–1.000), *p* < 0.001	0.757 (0.580–0.935), *p* = 0.020
Best day 1 OSI	0.989 (0.963–1.00), *p* < 0.001	0.793 (0.650–0.937), *p* = 0.003	0.852 (0.723–0.981), *p* = 0.001	0.748 (0.561–0.934), *p* = 0.025
Highest day 1 OSI	1.000 (1.000–1.00), *p* < 0.001	0.699 (0.531–0.866), *p* = 0.045	0.904 (0.804–1.000), *p* < 0.001	0.814 (0.655–0.973), *p* = 0.005
Mean day 1 OSI	0.978 (0.939–1.00), *p* < 0.001	0.753 (0.599–0.908), *p* = 0.011	0.870 (0.748–0.992), *p* = 0.001	0.770 (0.594–0.945), *p* = 0.015

AUROC, area under the receiver operator characteristics; PHT, pulmonary hypertension; HFOV, high frequency oscillatory ventilation; OI, oxygenation index; OSI, oxygen saturation index.

With regard to pulmonary hypertension, all of the first, best, highest and mean OI and OSI in day 1 except for the admission OSI (AUC = 0.683, *p* = 0.065) were predictive of pulmonary hypertension (Table 4). Additionally, all of the first 24-hour OIs and OSIs except for the best day 1 OI (AUC = 0.674, *p* = 0.108) were predictive of the need for rescue HFOV (Table 4). Concerning the patch repair during surgery, OI indices did not yield significant results with the highest AUROC value being 0.652 (*p* = 0.170). This contrasts with OSI values which demonstrated significant predictive potential with AUROC ranging from 0.748 (*p* = 0.025) to 0.814 (*p* = 0.005) (Table 4).

**Figure 5:**
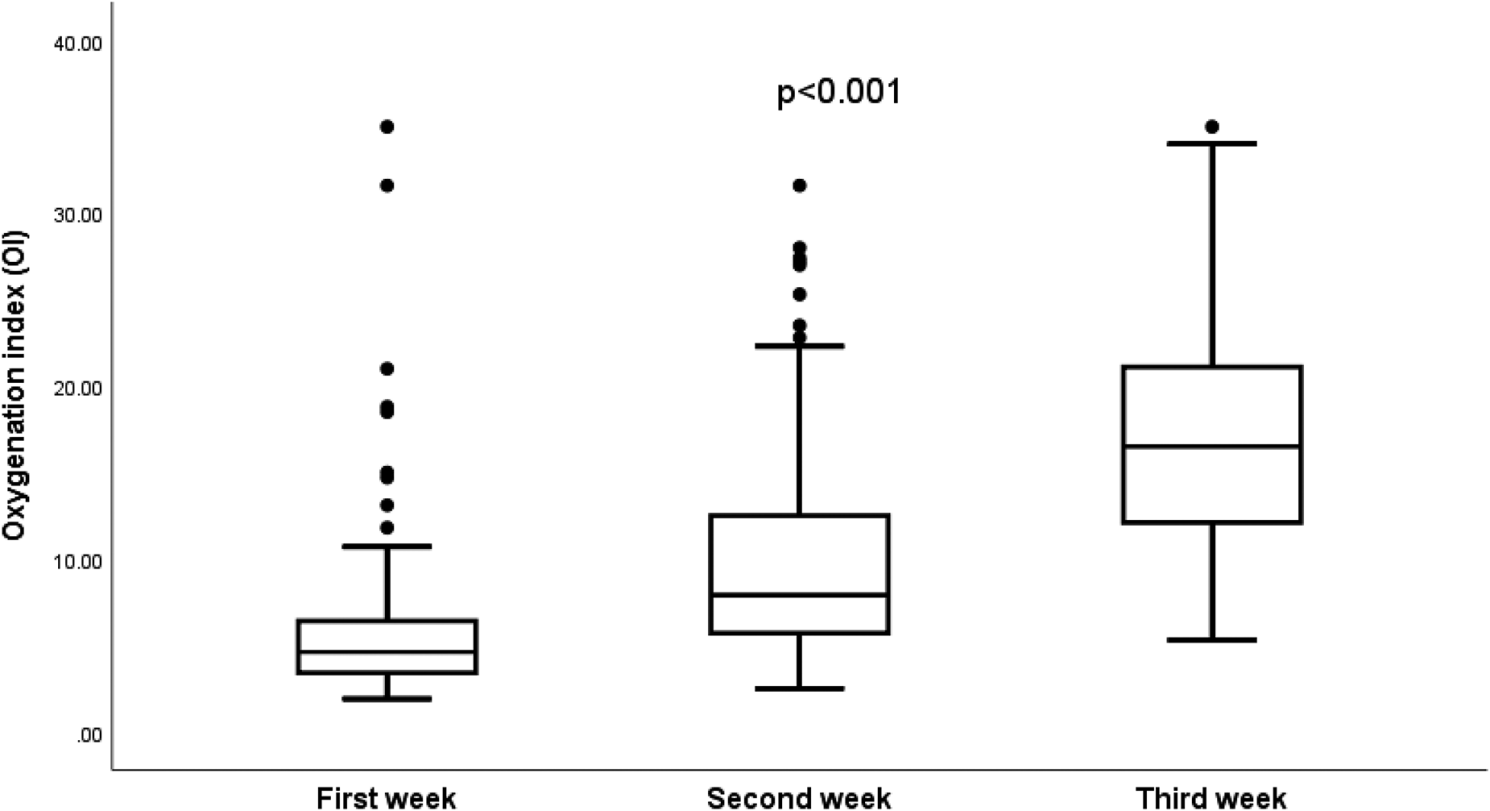
Trends of the oxygenation indices (OI) by the week of surgery.

In Figure 5, box plots depict the Oxygenation Index (OI) for the 29 infants who underwent surgery for the repair of congenital diaphragmatic hernia, based on the week of their surgery.

A noteworthy statistical difference is observed in the median oxygenation indices when comparing the three distinct infant groups, each grouped by their respective week of surgery (*p* < 0.001).

**Figure 6:**
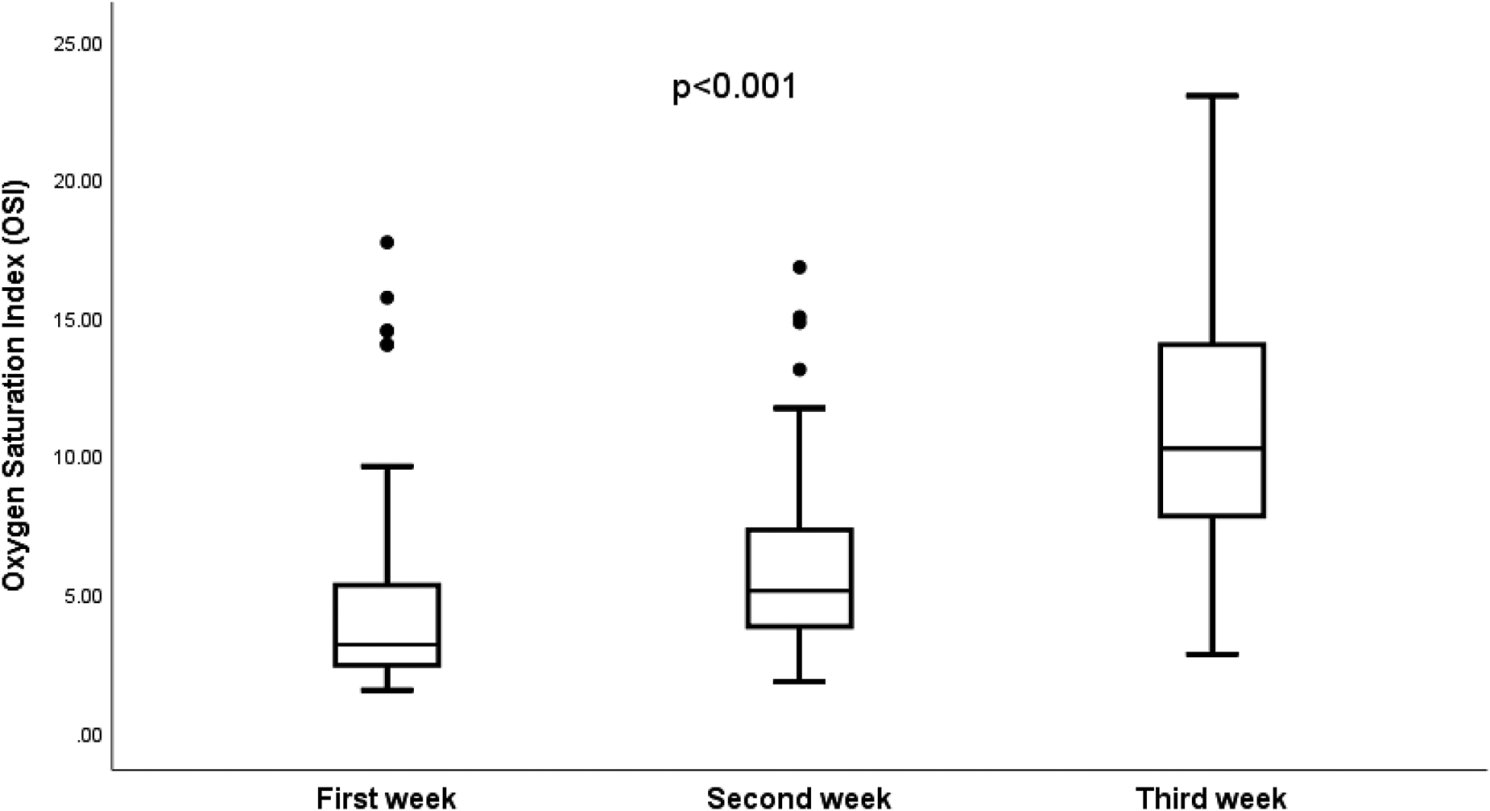
Trends of the oxygen saturation indices (OSI) by the week of s.

In Figure 6, the trends of Oxygen Saturation Indices (OSI) from birth to surgery are depicted for the 29 patients who survived to have surgery. The patients have been categorized based on the week they underwent surgical intervention for the diaphragmatic defect. A noteworthy statistical difference is observed in the median oxygen saturation indices when comparing the three distinct infant groups, each grouped by their respective week of surgery (*p* < 0.001).

## Discussion

The presented study focused in examining the relationship between OI and OSI in infants diagnosed with CDH from birth till the time of surgery. Moreover, wished to validate whether OSI can be a reliable predictor of survival and potential adverse neonatal outcomes in infants with CDH.

In our study involving 37 infants with CDH, we observed a robust correlation between OI and OSI values from birth up until the point of surgery or, unfortunately, death for those who didn't survive. Our research highlights a significant linear relationship between OSI, a non-invasive metric, and OI. We provide a regression formula to calculate OSI based on OI values. An in-depth examination across all OI/OSI pairs—whether matched or unmatched—revealed consistently derived OSI values relative to OI metrics. Specifically, by analyzing three distinct models (all pairs, matched pairs, and unmatched pairs of OI/OSI values), we determined that the clinical OI benchmarks of 10, 15, 20, and 40 were parallel to OSI values of 5, 8, 11, and 23, respectively.

The utilization of OSI is on the rise in both pediatric and adult intensive care units, serving as an indicator for respiratory failure and lung damage (15, 16). In a retrospective study involving 74 neonates, both late preterm and term, a significant correlation was observed between OSI and OI, especially when OI values were between 4 and 32 (5). Meanwhile, another prospective study of 54 neonates identified OSI values of 3 and 6.5 as corresponding to OI values of 5 and 15, respectively (17). More recently, a study focusing on OSI in neonates with CDH showed a strong correlation between the OI and OSI values determined within the first 24 h post-birth (11). The authors from that study deduced that for clinically pertinent OI values of 10, 15, 20, and 40, the respective OSI values were 9, 11, 13, and 21 (11). Significantly in the previous CDH OSI study, the OSI values derived from OI closely align with our findings, showcasing a consistency in observations across different CDH cohorts. A key distinction between our study and the prior research on OSI in CDH is the duration over which data was collected. Our models, which derived OSI from OI, were based on a broader set of OI/OSI pairs. This was because we recorded results over an extended duration from birth to the surgical intervention. In contrast, the earlier study focused specifically on values acquired within the initial 24 h post-birth. This extended data collection in our study offers a more comprehensive insight into the trends and correlations over time.

Moreover, our study showed that the OI and OSI values at the time of admission as well as highest, best, and mean values during the first 24 h post-birth were closely correlated, with the mean OI/OSI pairs showing the strongest correlation coefficient. While instantaneous readings, such as those taken at admission, highest, or best values, offer invaluable insights into the acute status of a patient's respiratory function, the mean OI/OSI over the initial 24 h paints a more comprehensive picture of the patient's respiratory status during this critical period. This is because the mean values of OI/OSI serves as an integrative measure of the respiratory function rather than just isolated measurements, which can be influenced by various transient factors. Additionally, we have demonstrated that these indices within the first 24 h predicted mortality as well as other adverse neonatal outcomes such as echocardiographic evidence of pulmonary hypertension and the need for rescue HFOV. A particularly compelling finding was that the OSI values within the first day, unlike the OI metrics, predicted the requirement for patch repair of diaphragmatic hernia—a procedure indicative of the magnitude of the diaphragmatic defect. It's worth noting, however, that our study lacked detailed quantitative data on the size of the diaphragmatic defect at the time of surgery. This limitation arises from the absence of consistent recording of this information in the surgical records of our patients.

Twenty-nine infants (78%) of the CDH infants in our study survived to undergo surgical repair of the diaphragmatic defect. We examined the patterns in oxygenation indices (OI) and oxygen saturation indices (OSI) contingent upon the surgical intervention's timing post-birth. We observed that infants who were operated on during the first week post-birth demonstrated comparatively lower indices than those who had surgery in the subsequent second and third weeks. Furthermore, a notable divergence in OI and OSI values was evident between infants operated on in the second week and those who had their surgeries in the third week. These discernible trends underscore the potential of OI and OSI as indicators in forecasting the requisite clinical stability for infants with CDH prior to surgical intervention. To the best of our knowledge, this study is the first to describe the OI and OSI trajectories in infants with CDH from their time of birth leading up to the time of surgery.

Our study has both distinct strengths and certain limitations. It distinguishes itself as only the second study reporting on the correlations between the OI and OSI in infants with CDH. Notably, to our knowledge, our research breaks new ground by analyzing the relationships between these indices beyond the immediate 24-hour postnatal period. Furthermore, our work sheds light on the predictive ability of OI and OSI values regarding a range of key neonatal outcomes, not just limited to survival rates. Finally, a significant contribution of our research is being the first to elucidate the trends of both the OI and OSI from birth leading up to the time of surgical intervention for repair of the diaphragmatic defect. Nevertheless, our study is not without its limitations. One of the limitations of our study is that we did not specifically address the potential plateau effect of the (OSI) values. When SpO_2_ reaches its maximum of 100%, OSI values can plateau, while Oxygenation Index (OI) can continue to increase. This limitation suggests the need for careful interpretation of OSI trajectories, particularly when SpO_2_ values approach 100%. Future studies should consider incorporating methods to account for this plateau effect. The retrospective nature of our investigation, while comprehensive in its data gathering approach, could inherently bring forth certain biases. Furthermore, the decision to measure arterial blood gases with subsequent measurement of OI was left to the clinician's judgment. We gathered information on the timing of arterial blood gas assessments and SpO_2_ recordings as near to the actual moment as feasible, according to the respiratory therapists' notes. By examining the electronic medical records, we deduced that the average time gap between these readings was likely under a minute. Additionally, a notable limitation of our study is the lack of systematically documented quantitative data on the size of the diaphragmatic defect. To address this gap, we used the need for patch repair as a surrogate marker for defect size, with 41% of our infants requiring patch repair during surgery. Patch repair is typically necessitated by larger defects, thus serving as an indirect indicator of defect size. While this approach provides a practical alternative for assessing the impact of defect size, it has its limitations and may not fully capture the variability in defect sizes. Finally, given that our institution neither offers Extracorporeal Membrane Oxygenation (ECMO) for infants diagnosed with CDH nor are such infants transferred for ECMO elsewhere, our findings cannot comment on the ability of OI and OSI in predicting the need for ECMO.

Based on our findings, we propose developing of software that integrates data on MAP, FiO_2_, and SpO_2_, and subsequently displays the OSI in real-time for mechanically ventilated CDH patients. Such an integrated system would provide clinicians the ability to instantaneously assess any changes in the respiratory status leading to swifter clinical decision-making and timely interventions. Given the strong correlations observed between OI and OSI in our study, the move toward this kind of technology-driven, would provide an avenue for uninterrupted, non-invasive monitoring of an infant's respiratory condition, potentially reducing the reliance on arterial blood sampling, thus enhancing the efficiency and safety of care.

## Conclusion

This study identified a strong correlation between OI and OSI from birth till the time of surgery among infants with CDH. The second major conclusion was that the non-invasive OSI measurement in infants with CDH can be used as a predictor for a range of key neonatal outcomes including mortality, echocardiographic evidence of pulmonary hypertension, need for rescue HFOV and patch repair of the diaphragmatic defect at surgery.

## Data Availability

The raw data supporting the conclusions of this article will be made available by the authors, without undue reservation.
